# A Homochiral Multifunctional Metal-Organic Framework with Rod-Shaped Secondary Building Units

**DOI:** 10.3390/nano7040088

**Published:** 2017-04-21

**Authors:** Kun Cai, Nian Zhao, Ning Zhang, Fu-Xing Sun, Qing Zhao, Guang-Shan Zhu

**Affiliations:** 1 State Key Laboratory of Inorganic Synthesis and Preparative Chemistry, Jilin University, Changchun 130012, China; caikun2010@gmail.com (K.C.); zhaonian0809@126.com (N.Z.); zhangning_jlu@163.com (N.Z.); 2 China-Japan Union Hospital, Jilin University, Changchun 130033, China; 3 Key Laboratory of Polyoxometalate Science of the Ministry of Education, Faculty of Chemistry, Northeast Normal University, Changchun 130024, China; zhugs@mail.jlu.edu.cn; 4 Key Laboratory for Micro-Nano Energy Storage and Conversion Materials of Henan Province, Institute of Surface Micro and Nano Materials, Xuchang University, Xuchang 461000, China

**Keywords:** metal-organic frameworks (MOFs), homochiral, camphoric acid, multifunction

## Abstract

A new homochiral multifunctional metal-organic framework, [Zn_2_(CTBA)_2_·H_2_O] (JUC-112), was synthesized under solvothermal conditions, through the design of chiral ligand 4-(3-carboxy-2,2,3-trimethylcyclopentanecarboxamido) benzoic acid (H_2_CTBA) based on camphoric acid as building block. The crystal structure of the new material is a 2-dimensional (2D) chiral layer packed with infinite rod-shaped secondary building units (SBUs). The homochiral framework was identified by circular dichroism (CD) spectrum. Thermogravimetric measurement indicates its high thermal stability up to 450 °C. In addition, JUC-112 exhibits the capability of separating water from alcohols, second-order nonlinear optical effect, and photoluminescence.

## 1. Introduction

Over the past two decades, metal-organic frameworks (MOFs) have attracted vast attention owing to their designable structures and potential applications for catalysis, gas storage and separation, sensing, and so on [1,2,3,4,5,6,7]. In addition, some of these features could be integrated into an individual, multifunctional MOFs. Recently, a large number of multifunctional MOFs have been successfully synthesized [8,9,10,11]. As identified in these papers, a functional organic ligand is a type of active centre in constructing multifunctional MOFs, and can be designed to obtain the target properties of MOFs [12]. Most of these ligands were created with an aromatic ring and functional sites, which can create an interesting structure, and the target MOFs proved to be widely used in selective sorption, luminescent sensors, and catalysis [13,14,15,16]. Among the ligands, only some of them were designed by introducing homochiral groups to construct homochiral structures, which could be potentially used in asymmetric catalysis, ferroelectric, and nonlinear optical applications [17,18,19,20]. It is also suggested that chiral structures with other functional groups would give more interesting properties for potential applications.

The chiral camphoric group has been proved to be able to effectively construct homochiral frameworks [21,22,23]. Herein, we attempt to extend the camphoric acid ligand with an aromatic ring, which may enhance the length of the ligand and the thermal stability of the framework [24], in order to synthesize the ligand 4-(3-carboxy-2,2,3-trimethylcyclopentanecarboxamido) benzoic acid (H_2_CTBA). At the same time, the attractive acylamide group in H_2_CTBA can be used as a guest-accessible functional organic site to produce the interactions between the framework and guest molecules [25,26,27,28]. The strategy of incorporating functional groups on the organic bridging ligands will be useful to obtain MOFs with multifunctional properties [29,30,31,32].

**Scheme 1:**
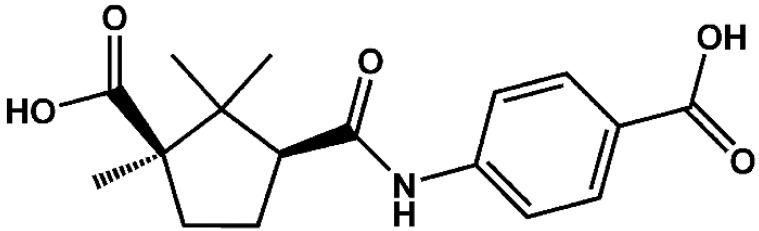
Molecular structure of the ligand (CTBA).

Hence, on the basis of the above consideration, the ligand H_2_CTBA based on d-(+)-camphoric acid is successfully prepared and used to synthesize MOFs (Scheme 1). The multifunctional homochiral MOFs [Zn_2_(CTBA)_2_·H_2_O]*_n_*·(JUC-112) constructed from CTBA^4−^ and Zn^2+^ is obtained by the solvothermal reaction of Zn(NO_3_)_2_·6H_2_O and H_2_CTBA in the solvent mixture of water and 2-butyl alcohol. Its structure can be deconstructed as a kind of MOFs with rod-shaped secondary building units (SBUs) linked by the ligand [33]. Furthermore, due to the existence of the multifunctional ligand, the properties of JUC-112 such as water-alcohol separation, luminescence and second-order nonlinear optical (NLO) effect are studied. 

## 2. Results and Discussion

### 2.1. Crystal Structure 

**Figure 1:**
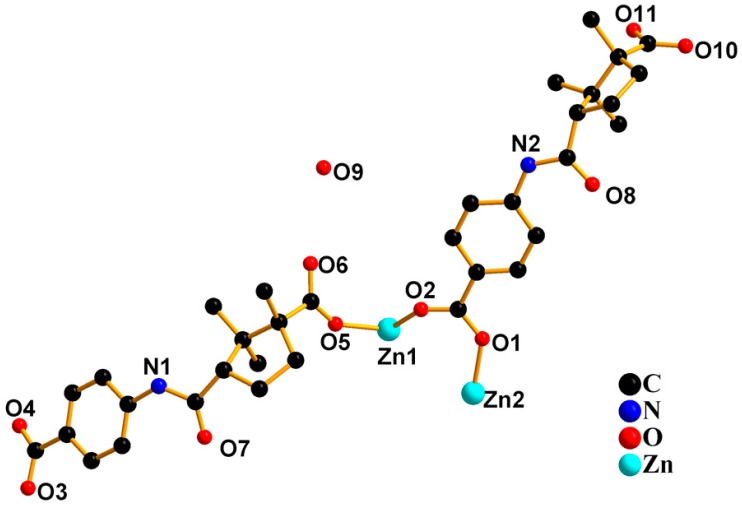
Asymmetric unit of JUC-112. The hydrogen atoms have been omitted for clarity.

**Figure 2:**
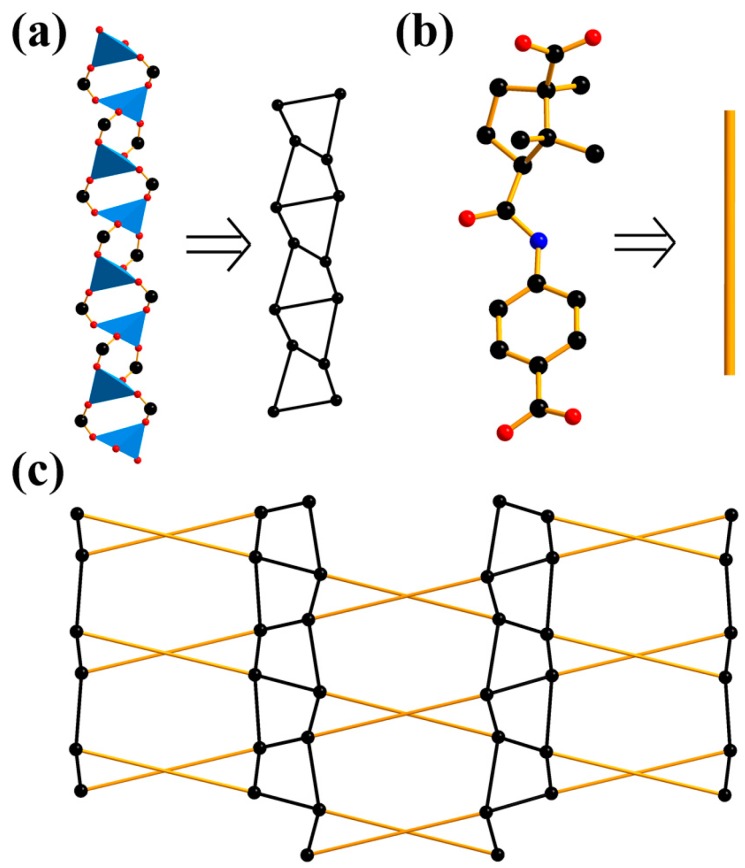
(**a**) Rod-shaped secondary building unit (SBU) with polyhedral Zn is simplified as a twisted ladder; (**b**) The ligand CTBA is considered as a stick. The hydrogen atoms have been omitted for clarity; (**c**) The net of the 2-dimensional layer framework.

Single crystal X-ray diffraction reveals that JUC-112 crystallizes in the chiral monoclinic *C*2 space group. The whole framework of JUC-112 shows a 2D chiral layer structure. Crystallographic data and details of refinements for JUC-112 are given in supporting information, Table S1. Its asymmetric unit contains two Zn^2+^ ions, two deprotonated ligands and one water molecule (Figure 1). One of the Zn^2+^ ions is four-coordinated by four O atoms of the carboxyl group moieties from four different ligands (Table S2, Zn1–O5 = 1.893(3) Å; Zn1–O4^#5^ = 1.923(2) Å; Zn1–O10^#3^ = 1.934(3) Å; Zn1–O2 = 1.926(3) Å), and the other Zn^2+^ ion takes the same coordination geometry. There are four independent carboxyl groups from the two ligands. They are coordinated to the metal ions in a bidentate mode. The zinc atoms are connected by the carboxyl groups to form an infinite ···Zn–O–C–O–Zn··· rod-shaped SBU. Two adjacent SBUs are connected by the ligands to form a 2D layer. Topology analysis is conducted by the software TOPOS 4.0. Each C atom in the carboxyl group moieties can be treat as the points of extension, so the rod-shape SBU could be simplified as a twisted ladder (Figure 2a). The ligand CTBA is ditopic and can be simplified as a stick (Figure 2b). These rod-shaped SBUs (twisted ladders) are linked with each other by these sticks to form the 2D chiral layer, which is a 4-c 2D network topology with point symbol for net is {4^3^·6^3^} (Figure 2c). This 2D structure constructed from Zn and H_2_CTBA is different from our previously reported 3-dimensional (3D) Cd compound with the same ligand [34], the reason for which can be interpreted with the higher coordination numbers of Cd than Zn.

**Figure 3:**
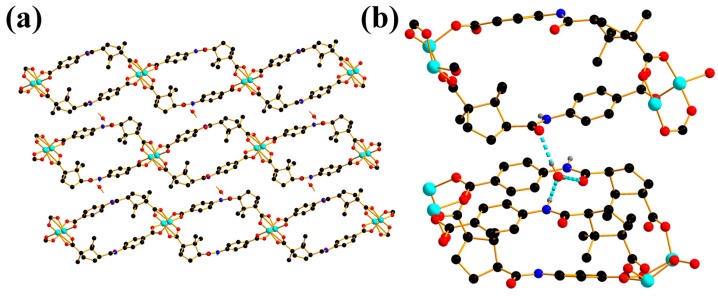
(**a**) The 3D framework of JUC-112; (**b**) The hydrogen bonds between the layers.

The intermolecular hydrogen bonds extend the structure from the 2D layer to the 3D supramolecular structure (Figure 3a). Figure 3b shows the interactions of hydrogen bonds between the layers. It can be seen that the water molecule interacts with the amide moieties of the adjacent layer by the hydrogen bond as a hydrogen bond donor (O–H···O = 1.95 Å, ∠DHA = 167.3°) and another layer by the hydrogen bonds as both a hydrogen bond donor (O–H···O = 2.09 Å, ∠DHA = 155.6°) and acceptor (N–H···O = 2.01 Å, ∠DHA = 177.6°).

### 2.2. Powder X-ray Diffraction Analysis and Thermogravimetric Analysis (TG) 

**Figure 4:**
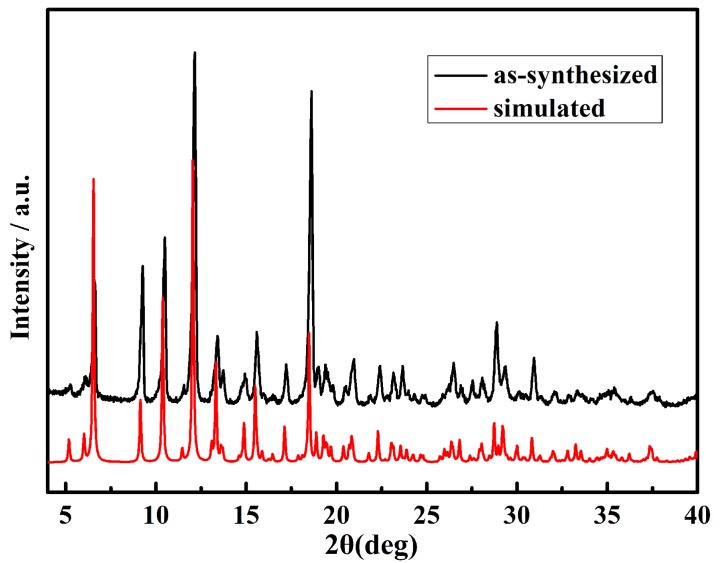
Powder X-ray diffraction of JUC-112.

In order to confirm the phase purity of the as-synthesized sample, a powder X-ray diffraction (PXRD) test is conducted. As shown in Figure 4, the peak positions are well coincident, with very little differences existing in some peaks’ intensities. The consistence between the experimental PXRD pattern and the simulated one validates that the crystal structure of JUC-112 is truly representative of the bulk material. 

**Figure 5:**
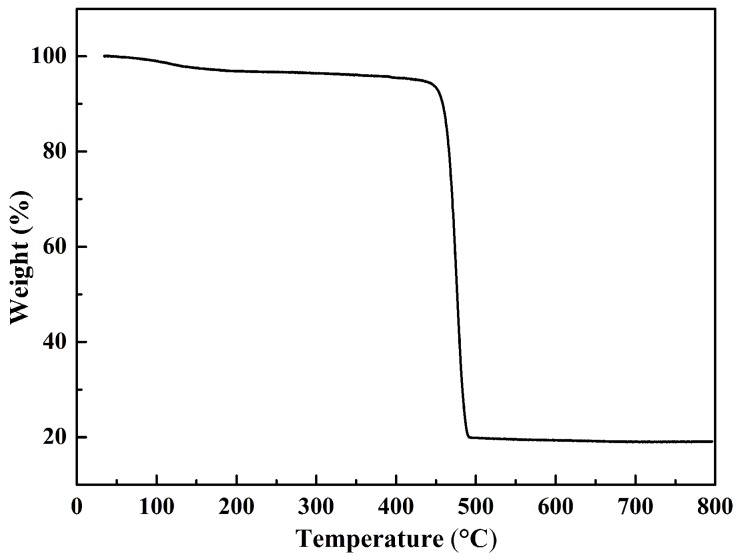
The thermogravimetric analysis (TG) curve of as-synthesized JUC-112.

The TG curve of crystalline sample of JUC-112 is measured under flowing air atmosphere in the temperature range of 35–800 °C. It shows a weight loss of 3.07% from 35 to 200 °C corresponding to the removal of H_2_O molecules (calcd. 2.29%), followed by a plateau (200–420 °C) indicating its high thermal stability (Figure 5). The framework is completely decomposed before 480 °C. The residue is supposed to be ZnO.

### 2.3. Circular Dichroism (CD) Spectrum

**Figure 6:**
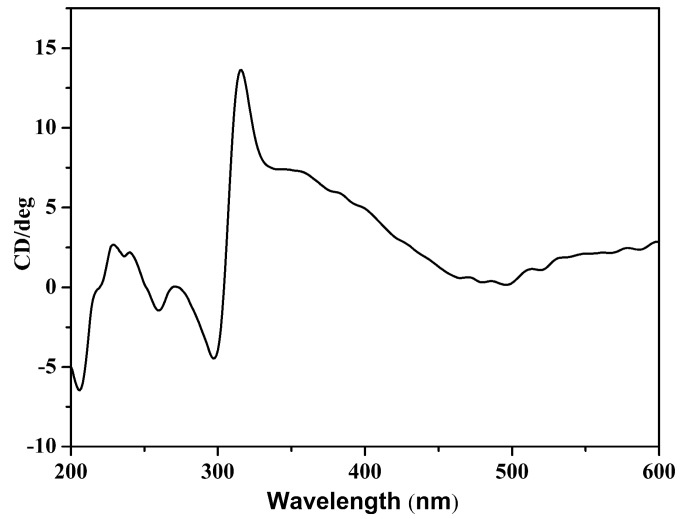
The solid-state circular dichroism (CD) spectra of JUC-112.

To further investigate the homochiral nature of JUC-112, solid-state circular dichroism (CD) measurements are performed on a KBr plate with solid material (KBr:JUC-112 = 50 mg:1 mg). As shown in Figure 6, it exhibits an obvious Cotton effect at 315 nm, which reveals that all the samples of JUC-112 crystallize in homochirality.

### 2.4. Gas and Vapor Sorption Study

**Figure 7:**
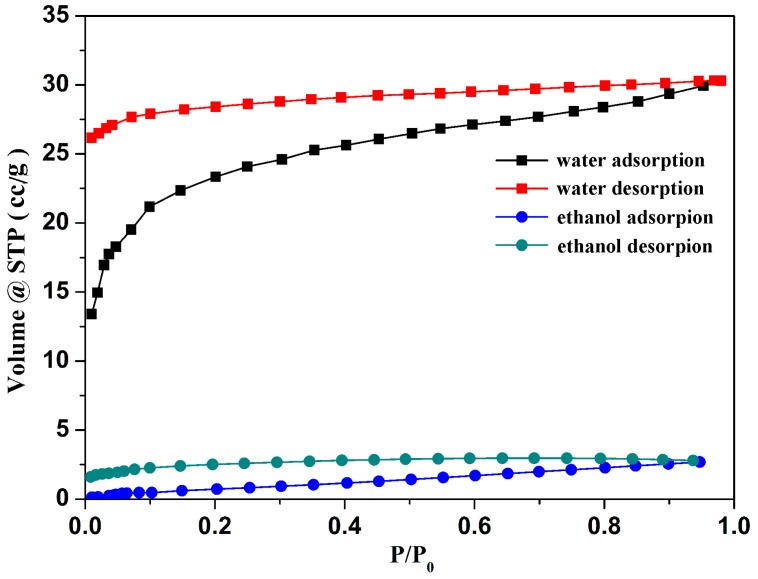
Water (squares) and ethanol (circles) vapor adsorption isotherms of JUC-112 at 298 K.

According to the crystal structure, we can see that JUC-112 has narrow channels in the direction [010] and amide groups in the wall of channels, which can absorb small guest molecules. However, the adsorption isotherms of N_2_ at 77 K and CO_2_ at 273 K reveal that almost no adsorptions are found, indicating that N_2_ and CO_2_ molecules (kinetic diameter 3.64 Å and 3.3 Å) could not be adsorbed [35]. To confirm the porous nature of the framework, vapor adsorption isotherms for water and ethanol are also measured at 298 K (Figure 7). The adsorption isotherm shows a hysteretic behavior, which may be caused by the interactions between guest molecules and –NH groups of the ligands [28]. JUC-112 exhibits hysteretic adsorption behavior to water (2.68 Å) with an uptake of 30 cc/g (P/P_0_ = 0.98). However, almost no uptake (2.95 cc/g, P/P_0_ = 0.98) for ethanol (4.5 Å) is found. These experimental results indicate that JUC-112 could accommodate water molecules, but exclude ethanol with a larger kinetic diameter. Thus, JUC-112 can size-selectively absorb water from alcohols, which indicates that it may be used as an adsorbent in the purification of alcohols from aqueous media. 

### 2.5. Second-Order NLO Effect

JUC-112 crystallizes in the chiral space group *C*2, so it theoretically exhibits the second-order NLO effect. The second harmonic generation (SHG) intensity of JUC-112 is estimated using the powder sample. The preliminary experimental result shows that JUC-112 indeed displays SHG activity with a value of approximately 0.5 times comparing with the urea. The SHG efficiency of JUC-112 is likely to result from the non-centrosymmetricity of the structure.

### 2.6. Photoluminescence

**Figure 8:**
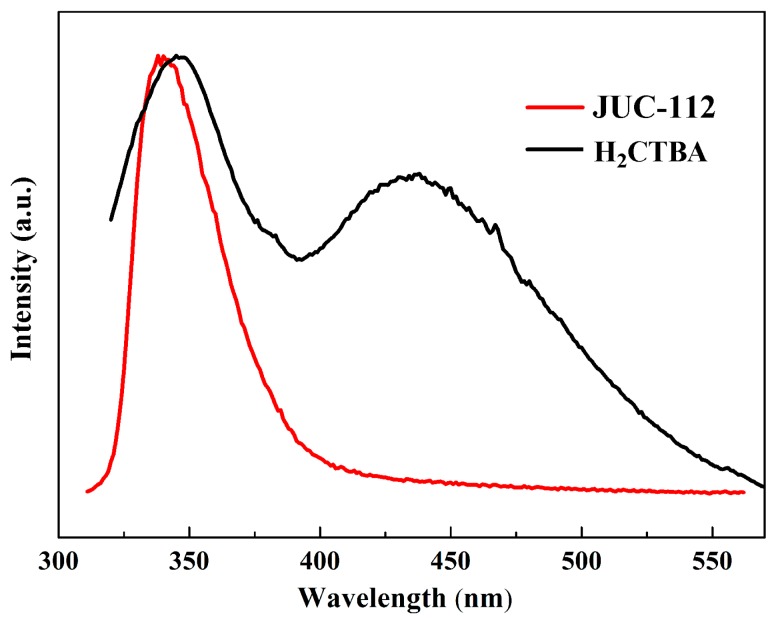
Solid-state luminescence emission spectra of JUC-112 and the ligand at room temperature.

The solid-state photoluminescence properties of JUC-112, as well as the free ligand, are studied at room temperature. The excitation peaks are obtained by the UV-Vis spectrum (Figure S1). The emission peak of the free ligand is observed at about 346 nm, with the excitation peak at 300 nm. JUC-112 shows an emission band at 340 nm under an excitation wavelength at 291 nm (Figure 8). Taking the similarity of the emission of JUC-112 to that of the ligand into account, the luminescence of JUC-112 is probably caused by the ligand-to-metal charge transfer (LMCT).

## 3. Materials and Methods 

All reagents and solvents were purchased from commercial sources without further purification. Powder X-ray diffractions (PXRD) are performed by a Riguku D/MAX2550 diffractometer (Shimadzu, Tokyo, Japan) using Cu-Kα radiation, 40 kV, 200 mA. The TG curve is obtained in air in the temperature range of 35–800 °C on a Netzch Sta 449c thermal analyzer (Netzch GmbH, Selb, Germany). The elemental analysis is carried out on a Perkin-Elmer 240C elemental analyzer (PerkinElmer, Waltham, MA, USA). The CD spectrum is obtained on a Biologic MOS-450 (Bio-Logic, Seyssinet-Pariset, France) by the pellets of the crystals. Pellets of JUC-112 with a 6-mm diameter and a thickness of 0.25–0.3 mm were prepared by pressing the powder material at 40 kN cm^−2^. Photoluminescence property is performed on a Perkin-Elmer LS55 luminescence spectrometer (PerkinElmer, Waltham, MA, USA). Organic vapor and gas sorption isotherms are measured on the Autosorb-iQ2-AG-VP adsorption analyzer (Quantachrome, Boynton Beach, FL, USA). A pulsed Q-switched Nd:YAG laser (Nanjing University, Nanjing, China) at a wavelength of 1064 nm is used to generate an SHG signal from powder samples. The backscattered SHG light is collected by a spherical concave mirror and passes through a filter that transmits only 532 nm radiation.

Synthesis of the ligand. The ligand is synthesized according to the literature [34]. (+)-Camphoric acid (4.0 g, 20 mmol), acetic anhydride (12 mL) are added to a flask. The mixture is stirred for 4 h with reflux. The resulting solution is cooled down to room temperature and kept still overnight. Colorless crystalline powder of (+)-camphoric anhydride is obtained after filtration and drying. (+)-Camphoric anhydride (2.389 g, 13 mmol), 4-aminobenzoic acid (1.795 g, 13 mmol) and anhydrous sodium acetate (1.074 g, 13 mmol) are added to a mortar for uniform grind. Then quartz sand (18 g) are added and mixed uniformly. The mixture is stirred at 145 °C for 4 h. After cooling to room temperature, ethanol/water mixture (80 mL/20 mL) is added for filtering the quartz sand. The filtered solution is partially eliminated under reduced pressure. White precipitate is filtered after adding water and stirring for 12 h (yield 70%).

Synthesis of Zn_2_(C_17_H_19_NO_5_)_2_·H_2_O (JUC-112). A mixture of ligand (24 mg, 0.075 mmol), Zn(NO_3_)_2_·6H_2_O (22 mg, 0.075 mmol), water (300 μL) and BuOH (700 μL) is sealed in the vacuous glass tube and heated at 120 °C for 2 days. Colorless flake crystals of JUC-112 are obtained in a 42% yield based on Zn. Elemental analysis: calc. C, 52.11; H, 5.11; N, 3.58. Found: C, 52.29; H, 5.05; N, 3.40. 

X-ray crystallography. Crystallographic data of the compound is collected at 293 K on Bruker Apex II CCD area-detector diffractometer (Mo-Kα, 0.71073 Å, Bruker, Karlsruhe, Germany). The structure is solved with direct methods and refined by the full matrix least-squares method on F2 using *SHELXL*-2014. Anisotropic `thermal parameters are applied to all the non-hydrogen atoms. Hydrogen atoms are fixed at calculated positions and refined by using the riding mode. Crystallographic data are summarized in Table S1; the selected bond lengths and bond angles of JUC-112 are listed in Table S2. Hydrogen bonds for the crystal are shown in Table S3. The crystal CCDC Number: 915365.

## 4. Conclusions

In summary, the homochiral metal-organic framework JUC-112 has been successfully synthesized using a newly designed ligand. This 2D MOF is composed of interesting infinite rod-shaped building blocks, and exhibits multifunctional properties (alcohol/water separation, second-order nonlinear optical effect and photoluminescence) owing to the chirality and amide groups. Our target is to join the functional groups together for broader application prospects. To obtain the multifunctional chiral porous frameworks is still challenging. Further studies on the functional groups of chiral ligands are still under way in our laboratory. 

## Supplementary Materials

The following are available online at http://www.mdpi.com/2079-4991/7/4/88/s1. Figure S1: UV-visible spectrum of the ligand and JUC-112, Table S1: Crystallographic data and details of refinements for JUC-112, Table S2: Selected bond lengths [Å] and angles [deg] for JUC-112, Table S3: Hydrogen bonds for JUC-112 [Å and °].
