# Supplementary material for: A Homochiral Multifunctional Metal-Organic Framework with Rod-Shaped Secondary Building Units
Source: Nanomaterials (Basel). 2017 Apr 21;7(4):88. doi: 10.3390/nano7040088 (PMC5408180; doi:10.3390/nano7040088)

# checkCIF/PLATON report

You have not supplied any structure factors. As a result the full set of tests cannot be run.

THIS REPORT IS FOR GUIDANCE ONLY. IF USED AS PART OF A REVIEW PROCEDURE FOR PUBLICATION, IT SHOULD NOT REPLACE THE EXPERTISE OF AN EXPERIENCED CRYSTALLOGRAPHIC REFEREE.

No syntax errors found.    CIF dictionary    Interpreting this report

## Datablock: I1

---

```
Bond precision:  C-C = 0.0082 A          Wavelength=0.71073

Cell:           a=31.0626(16)    b=6.8540(4)        c=19.5825(11)
                alpha=90         beta=119.507(3)    gamma=90
Temperature:    293 K

                Calculated                  Reported
Volume          3628.4(4)                   3628.4(4)
Space group     C 2                         C 2
Hall group      C 2y                        C 2y
Moiety formula  C34 H38 N2 O10 Zn2, H2 O    ?
Sum formula     C34 H40 N2 O11 Zn2          C34 H40 N2 O11 Zn2
Mr              783.46                      783.46
Dx,g cm-3       1.434                       1.434
Z               4                           4
Mu (mm-1)       1.382                       1.382
F000            1624.0                      1624.0
F000’           1626.98
h,k,lmax        41,9,26                     41,9,26
Nref            9083[ 4914]                 8333
Tmin,Tmax       0.745,0.780                 0.864,0.864
Tmin’           0.731

Correction method= # Reported T Limits: Tmin=0.864 Tmax=0.864
AbsCorr = MULTI-SCAN

Data completeness= 1.70/0.92      Theta(max)= 28.381

R(reflections)= 0.0396( 5925)     wR2(reflections)= 0.0791( 8333)

S = 0.982                  Npar= 449
```

---

The following ALERTS were generated. Each ALERT has the format
**test-name_ALERT_alert-type_alert-level**.
Click on the hyperlinks for more details of the test.

## Alert level C

```
PLAT241_ALERT_2_C High   'MainMol' Ueq as Compared to Neighbors of         O1 Check
PLAT241_ALERT_2_C High   'MainMol' Ueq as Compared to Neighbors of         O2 Check
PLAT241_ALERT_2_C High   'MainMol' Ueq as Compared to Neighbors of         O5 Check
PLAT241_ALERT_2_C High   'MainMol' Ueq as Compared to Neighbors of        C15 Check
PLAT241_ALERT_2_C High   'MainMol' Ueq as Compared to Neighbors of        C22 Check
PLAT242_ALERT_2_C Low    'MainMol' Ueq as Compared to Neighbors of        Zn1 Check
PLAT242_ALERT_2_C Low    'MainMol' Ueq as Compared to Neighbors of        C20 Check
PLAT242_ALERT_2_C Low    'MainMol' Ueq as Compared to Neighbors of        C21 Check
PLAT341_ALERT_3_C Low Bond Precision on  C-C Bonds ...............    0.00821 Ang.
PLAT352_ALERT_3_C Short   N-H (X0.87,N1.01A)  N1     -   H36    ..       0.74 Ang.
PLAT420_ALERT_2_C D-H Without Acceptor       N1     --  H36    ...     Please Check
PLAT480_ALERT_4_C Long H...A H-Bond Reported H19    ..  O9      ..       2.68 Ang.
PLAT480_ALERT_4_C Long H...A H-Bond Reported H12    ..  O7      ..       2.66 Ang.
PLAT601_ALERT_2_C Structure Contains Solvent Accessible VOIDS of .         39 Ang3
```

## Alert level G

```
PLAT004_ALERT_5_G Polymeric Structure Found with Maximum Dimension          2 Info
PLAT007_ALERT_5_G Number of Unrefined Donor-H Atoms ..............          4 Report
PLAT128_ALERT_4_G Alternate Setting for Input Space Group  C2              I2 Note
PLAT199_ALERT_1_G Reported _cell_measurement_temperature ..... (K)        293 Check
PLAT200_ALERT_1_G Reported   _diffrn_ambient_temperature ..... (K)        293 Check
PLAT791_ALERT_4_G The Model has Chirality at C13     (Chiral SPGR)          S Verify
PLAT791_ALERT_4_G The Model has Chirality at C16     (Chiral SPGR)          R Verify
PLAT791_ALERT_4_G The Model has Chirality at C19     (Chiral SPGR)          S Verify
PLAT791_ALERT_4_G The Model has Chirality at C21     (Chiral SPGR)          R Verify
```

0 **ALERT level A** = Most likely a serious problem - resolve or explain
0 **ALERT level B** = A potentially serious problem, consider carefully
14 **ALERT level C** = Check. Ensure it is not caused by an omission or oversight
9 **ALERT level G** = General information/check it is not something unexpected

2 ALERT type 1 CIF construction/syntax error, inconsistent or missing data
10 ALERT type 2 Indicator that the structure model may be wrong or deficient
2 ALERT type 3 Indicator that the structure quality may be low
7 ALERT type 4 Improvement, methodology, query or suggestion
2 ALERT type 5 Informative message, check

It is advisable to attempt to resolve as many as possible of the alerts in all categories. Often the minor alerts point to easily fixed oversights, errors and omissions in your CIF or refinement strategy, so attention to these fine details can be worthwhile. In order to resolve some of the more serious problems it may be necessary to carry out additional measurements or structure refinements. However, the purpose of your study may justify the reported deviations and the more serious of these should normally be commented upon in the discussion or experimental section of a paper or in the "special_details" fields of the CIF. checkCIF was carefully designed to identify outliers and unusual parameters, but every test has its limitations and alerts that are not important in a particular case may appear. Conversely, the absence of alerts does not guarantee there are no aspects of the results needing attention. It is up to the individual to critically assess their own results and, if necessary, seek expert advice.

**Publication of your CIF in IUCr journals**

A basic structural check has been run on your CIF. These basic checks will be run on all CIFs submitted for publication in IUCr journals (*Acta Crystallographica*, *Journal of Applied Crystallography*, *Journal of Synchrotron Radiation*); however, if you intend to submit to *Acta Crystallographica Section C* or *E* or *IUCrData*, you should make sure that full publication checks are run on the final version of your CIF prior to submission.

**Publication of your CIF in other journals**

Please refer to the *Notes for Authors* of the relevant journal for any special instructions relating to CIF submission.

---

**PLATON version of 26/02/2017; check.def file version of 21/02/2017**

**Datablock I1** - ellipsoid plot

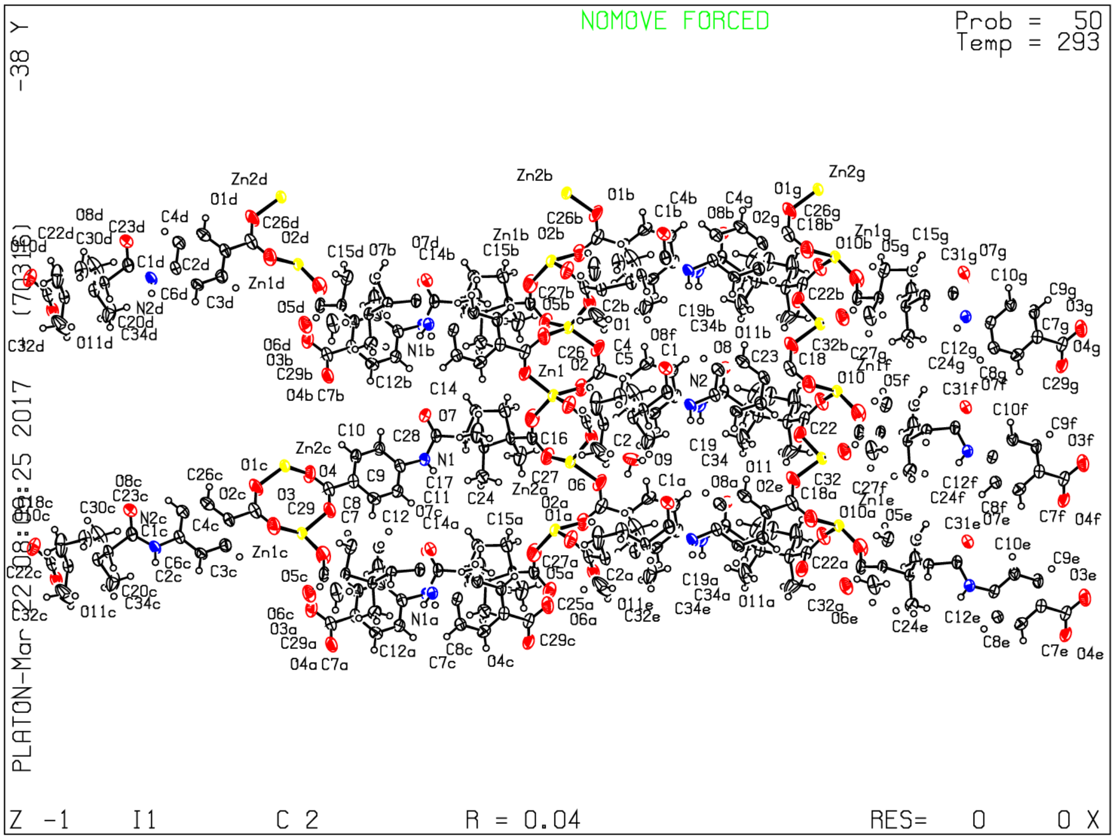

Supplement: Supplementary file 1 [file nanomaterials-07-00088-s001.zip › nanomaterials-189071-proof/checkcif.pdf]
