# Supplementary material for: A Homochiral Multifunctional Metal-Organic Framework with Rod-Shaped Secondary Building Units
Source: Nanomaterials (Basel). 2017 Apr 21;7(4):88. doi: 10.3390/nano7040088 (PMC5408180; doi:10.3390/nano7040088)
Supplement: Supplementary file 1 [file nanomaterials-07-00088-s001.zip › nanomaterials-189071-proof/Supporting information--proof.docx]

A homochiral multifunctional metal-organic framework with rod-shaped secondary building units

Kun Cai^1^, Nian Zhao^1^, Ning-Zhang^1^, Fu-Xing Sun*^1^ Qing Zhao*^2^ and Guang-Shan Zhu ^3,4^

^1^ State Key Laboratory of Inorganic Synthesis and Preparative Chemistry, Jilin University, Changchun 130012, China; caikun2010@gmail.com (K.C); zhaonian0809@126.com (N.Z); zhangning_jlu@163.com (N.Z)

^2^ China-Japan Union Hospital, Jilin University, Changchun 130033, China; qingzhao888@hotmail.com (Q.Z).

^3^ Key Laboratory of Polyoxometalate Science of the Ministry of Education, Faculty of Chemistry, Northeast Normal University, Changchun 130024, China; zhugs@mail.jlu.edu.cn (G.S.Z).

^4^ Key Laboratory for Micro-Nano Energy Storage and Conversion Materials of Henan Province, Institute of Surface Micro and Nano Materials, Xuchang University, Xuchang 461000, China.

***** Correspondence: fxsun@jlu.edu.cn (F.X.S); qingzhao888@hotmail.com (Q.Z); Tel.: +86-431-85168887, Fax: +86-431-8516-8331 (F.X.S).





Figure S1. UV-visible spectrum of the ligand and JUC-112

Table S1. Crystallographic data and details of refinements for JUC-112

Identification code I1

Empirical formula C34 H40 N2 O11 Zn2

Formula weight 783.46

Temperature 293(2) K

Wavelength 0.71073 Å

Crystal system Monoclinic

Space group C2

Unit cell dimensions a = 31.0626(16) Å α= 90°.

b = 6.8540(4) Å β= 119.507(3)°.

c = 19.5825(11) Å γ = 90°.

Volume 3628.4(4) Å3

Z 4

Density (calculated) 1.434 Mg/m3

Absorption coefficient 1.382 mm-1

F(000) 1624

Crystal size 0.220 x 0.200 x 0.180 mm3

Theta range for data collection 1.507 to 28.381°.

Index ranges -41<=h<=29, -9<=k<=9, -14<=l<=26

Reflections collected 11543

Independent reflections 8333 [R(int) = 0.0255]

Completeness to theta = 25.242° 99.9 %

Absorption correction Semi-empirical from equivalents

Refinement method Full-matrix least-squares on F2

Data / restraints / parameters 8333 / 1 / 449

Goodness-of-fit on F2 0.982

Final R indices [I>2sigma(I)] R1 = 0.0396, wR2 = 0.0695

R indices (all data) R1 = 0.0668, wR2 = 0.0791

Absolute structure parameter -0.003(15)

Extinction coefficient n/a

Largest diff. peak and hole 0.243 and -0.355 e.Å-3

| Table S2. Selected Bond lengths [Å] and angles [deg] for JUC-112. |
| --- |
| \| O(1)-Zn(2) \| 1.921(2) \| O(11)-Zn(2)#4 \| 1.925(3) \| \| --- \| --- \| --- \| --- \| \| O(2)-Zn(1) \| 1.926(3) \| Zn(1)-O(4)#5 \| 1.923(2) \| \| O(3)-Zn(2)#1 \| 1.947(3) \| Zn(1)-O(10)#3 \| 1.934(3) \| \| O(4)-Zn(1)#1 \| 1.923(2) \| Zn(2)-O(11)#6 \| 1.925(3) \| \| O(5)-Zn(1) \| 1.893(3) \| Zn(2)-O(6)#7 \| 1.946(3) \| \| O(6)-Zn(2)#2 \| 1.946(3) \| Zn(2)-O(3)#5 \| 1.947(3) \| \| O(10)-Zn(1)#3 \| 1.934(3) \|  \|  \| \|  \|  \|  \|  \| \| O(5)-Zn(1)-O(4)#5 \| 95.82(12) \| O(1)-Zn(2)-O(11)#6 \| 94.56(12) \| \| O(5)-Zn(1)-O(2) \| 100.78(14) \| O(1)-Zn(2)-O(6)#7 \| 107.35(12) \| \| O(4)#5-Zn(1)-O(2) \| 127.07(12) \| O(11)#6-Zn(2)-O(6)#7 \| 129.33(12) \| \| O(5)-Zn(1)-O(10)#3 \| 125.33(15) \| O(1)-Zn(2)-O(3)#5 \| 126.95(13) \| \| O(4)#5-Zn(1)-O(10)#3 \| 102.63(12) \| O(11)#6-Zn(2)-O(3)#5 \| 97.91(11) \| \| O(2)-Zn(1)-O(10)#3 \| 107.38(12) \| O(6)#7-Zn(2)-O(3)#5 \| 103.56(11) \| |

Symmetry transformations used to generate equivalent atoms:

#1 -x,y-1,-z+1 #2 x,y-1,z #3 -x+1,y,-z+2 #4 -x+1,y-1,-z+2 #5 -x,y+1,-z+1

#6 -x+1,y+1,-z+2 #7 x,y+1,z

Table S3. Hydrogen bonds for JUC-112 [Å and °]

____________________________________________________________________________

D-H...A d(D-H) d(H...A) d(D...A) <(DHA)

____________________________________________________________________________

O(9)-H(38)...O(7)#8 0.84 2.10 2.882(5) 155.6

O(9)-H(37)...O(8)#9 0.82 1.96 2.770(5) 167.3

N(2)-H(35)...O(9)#10 0.82 2.01 2.832(6) 177.6

C(19)-H(19)...O(9)#10 0.98 2.68 3.486(6) 140.0

C(12)-H(12)...O(7)#2 0.93 2.66 3.474(6) 146.1

C(10)-H(10)...O(7) 0.93 2.41 2.934(6) 115.5

C(1)-H(1)...O(8) 0.93 2.32 2.891(6) 119.3

____________________________________________________________________________

Symmetry transformations used to generate equivalent atoms:

#1 -x,y-1,-z+1 #2 x,y-1,z #3 -x+1,y,-z+2

#4 -x+1,y-1,-z+2 #5 -x,y+1,-z+1 #6 -x+1,y+1,-z+2

#7 x,y+1,z #8 x+1/2,y-1/2,z #9 -x+1,y-1,-z+1

#10 -x+1,y,-z+1
